# Shortened Taper Duration after Implementation of a Standardized Protocol for Iatrogenic Benzodiazepine and Opioid Withdrawal in Pediatric Patients: Results of a Cohort Study

**DOI:** 10.1097/pq9.0000000000000079

**Published:** 2018-05-18

**Authors:** Jane M. Vipond, Amy L. Heiberger, Paul A. Thompson, Jody N. Huber

**Affiliations:** From the * Department of Pharmaceutical Services, Sanford USD Medical Center, Sioux Falls, S.Dak.; † Department of Pharmacy Practice, South Dakota State University College of Pharmacy and Allied Health Professions, Brookings, S.Dak.; ‡ University of South Dakota, Sanford School of Medicine, Vermillion, S.Dak.; § Departments of Pediatrics and Internal Medicine, Sanford USD Medical Center, Sioux Falls, S.Dak.; ¶ Department of Pediatrics, Sanford Children’s Hospital, Sioux Falls, S.Dak.

## Abstract

**Introduction:**

Methadone and lorazepam prescribing discrepancies for the use of iatrogenic withdrawal were observed among providers. A standardized pharmacist-managed methadone and lorazepam taper protocol was implemented at a pediatric tertiary care facility with the aim to reduce the length of taper for patients with iatrogenic withdrawal.

**Methods:**

A multidisciplinary team of nurses, pharmacists, and physicians reviewed the current literature, then developed and implemented a standardized withdrawal taper protocol. Outcomes were compared with a retrospective control group using past prescribing practices. The primary endpoint was the length of methadone and/or lorazepam taper. Secondary endpoints included evaluation for significant differences between the control and standardized protocol groups regarding additional breakthrough withdrawal medications, pediatric intensive care unit (PICU) and hospital length of stay. We also evaluated provider satisfaction with the protocol.

**Results:**

The standardized protocol group included 25 patients who received methadone and/or lorazepam taper. A retrospective control group contained 24 patients. Median methadone taper length before protocol implementation was 9.5 days with an interquartile range (IQR) of 5.5–14.5 days; after protocol implementation, it was 6.0 (IQR, 3.0–9.0) days (*P* = 0.0145). Median lorazepam taper length before protocol implementation was 13.0 (IQR, 8.0–18.0) days; after protocol implementation, it was 6.0 (4.0–7.0) days (*P* = 0.0006). A statistical difference between PICU length of stay, hospital length of stay, or the number of additional medications for breakthrough withdrawal was not found.

**Conclusions:**

The use of a standardized withdrawal protocol resulted in shorter taper duration for both the methadone and lorazepam groups. There was no difference in PICU or hospital length of stay.

## Abstract 2

Supplemental Digital Content is available in the text.

## INTRODUCTION

In the pediatric intensive care unit (PICU), critically ill children often require intubation. These children commonly receive medications including opioids and/or benzodiazepines for sedation, anxiolysis, and analgesia. Prolonged use of these medications can lead to iatrogenic withdrawal upon discontinuation.^1^ In a study by Katz et al.,^2^ pediatric patients who received at least 5 days of continuous infusion of opioids had a greater than 50% risk for iatrogenic withdrawal, whereas those who received greater than 9 days of continuous infusion had a 100% risk for iatrogenic withdrawal. More recent studies have found that higher cumulative dose, longer duration of infusion, and both process and system-level factors contribute to increased risk of opioid and benzodiazepine withdrawal.^3–6^ Medications frequently used to manage iatrogenic opioid and benzodiazepine withdrawal include methadone and lorazepam, respectively. Methadone, an opioid analgesic, has a prolonged half-life of 8–59 hours, with a mean half-life of 19 hours in pediatric patients.^7,8^ Iatrogenic benzodiazepine withdrawal is often managed with lorazepam, an intermediate-acting benzodiazepine with a half-life of approximately 17 hours in pediatric patients.^9^

Many authors have reported the use of methadone for the management of iatrogenic opioid withdrawal. However, there is no consensus among clinicians or published literature regarding the rate and frequency of taper.^10,11^ Previous studies have recommended tapering by 10–20% daily or every other day based on the patient’s risk for withdrawal.^12,13^ Furthermore, a recent study by Steineck et al.^14^ demonstrated that a pharmacist-managed approach to iatrogenic opioid withdrawal provides a shorter length of withdrawal treatment. Although methadone tapering is well described in the literature, there remains a lack of data regarding the use of lorazepam for iatrogenic benzodiazepine withdrawal.

We observed variability among providers in opioid and benzodiazepine dosing and length of withdrawal tapers. Additional concerns included the arbitrary use of withdrawal tapers and prolonged length of taper and the potential for increased length of stay. A multidisciplinary team of pharmacists, physicians, and nurses hypothesized that a standardized, pharmacist-driven methadone and lorazepam withdrawal taper protocol would result in shorter withdrawal tapers, decreased hospital length of stay, and increased provider satisfaction.

## MATERIALS AND METHODS

This project was a prospective cohort study comparing the standardized taper group to a correlative retrospective control group. We conducted the study in a 12-bed PICU located within a 148-bed pediatric tertiary care facility. The PICU averages 775 yearly admissions consisting mainly of medical/surgical patients. The local institutional review board approved the study design as a quality improvement project and waived the need for informed consent.

### Protocol

The study protocol, modified from Steineck et al.,^14^ was created by a group of pharmacists and critical care physicians after a literature review and consultation with additional pediatric institutions regarding dosing and scheduling for the taper.^2,12–24^ The complete protocol is available for review as **Supplemental Digital Content**, available at http://links.lww.com/PQ9/A23. All pediatric intensivists at this institution were in agreement to use the standardized protocol. The critical care nurses assessed patients for withdrawal symptoms every 12 hours using the Withdrawal Assessment Tool-1 (WAT-1).^1^ We implemented the WAT-1 tool simultaneously with the study protocol. Study protocol implementation occurred on January 5, 2015.

We based patient risk stratification on duration of opioid and benzodiazepine infusions as defined previously.^14^ Four strata were identified: low risk for patients who received less than 5 days of an infusion; moderate risk for those receiving 5–9 days of infusion; high risk for those receiving 10–27 days of infusion or cumulative morphine dose of 60–100 mg/kg or equivalent; and very high risk for patients receiving greater than or equal to 28 days of infusion or cumulative morphine dose of greater than or equal to 100 mg/kg or equivalent (**see Supplemental Digital Content**). As part of the protocol, providers prescribed standard doses of intravenous lorazepam and morphine for breakthrough withdrawal symptoms.

### Patient Selection

All patients receiving continuous opioid and/or benzodiazepine infusions in the PICU between January 5, 2015, and February 29, 2016, comprised the standardized protocol cohort for this study. This cohort was a convenience sample that we continued until the number of standardized protocol patients equaled the control group. If a patient was deemed at risk for withdrawal by the primary physician or based on WAT-1 scoring, the pharmacy team was consulted to evaluate for the use of the standardized withdrawal taper protocol. Dosing and withdrawal taper length were determined based on risk stratification. If the enrolled patient had symptoms of withdrawal throughout the taper according to WAT-1 scoring, a pharmacist reviewed the taper and adjusted if necessary.

We performed a retrospective chart review for comparison data. All patients who received methadone and/or lorazepam taper(s) for iatrogenic withdrawal between January 1, 2014, and April 30, 2014, were included in this control group. This period was chosen for its seasonal association with a high census and increased incidence of withdrawal tapers. We collected identical data for both groups.

### Data Collection

We collected demographic variables (ie, age, sex, diagnosis, length of intubation in days, length of continuous sedation in days). Additional data collected included length of taper in days, additional breakthrough medications (intravenous morphine and/or lorazepam as documented in the medication administration record); WAT-1 scoring (collected only for the standardized protocol group due to timing of WAT-1 implementation); oversedation requiring antidote (naloxone and/or flumazenil); length of PICU stay, and length of hospital stay. We also recorded patients who were discharged home on methadone and/or lorazepam withdrawal tapers.

Provider satisfaction was based on surveys before and after the introduction of the withdrawal taper protocol. Survey questions were graded using a 5-point Likert scale and evaluated satisfaction, time consumption, confusion and/or difficulty with implementation, appropriately meeting patient needs, and whether the method was beneficial.

### Endpoints

The primary endpoint was total duration of taper. Secondary endpoints included the number of additional medications administered for breakthrough withdrawal, PICU length of stay, hospital length of stay, incidences of patient oversedation, and provider satisfaction.

### Statistical Analysis

We calculated descriptive statistics and performed appropriate tests of differences. All data analyses were performed in SAS 9.4 (SAS Institute Inc., Cary, N.C.). For categorical variables, we calculated frequencies and percentiles. We performed comparisons between control and standardized protocol cohorts using tests of proportions (Fisher exact tests and χ^2^ tests, PROC FREQ in SAS). For continuous time variables (which can be positively skewed), we divided the data into 4 equal parts and calculated the medians and the interquartile ranges (IQR). Comparisons were made between control and standardized protocol cohorts using median tests (PROC NPAR1WAY in SAS). For other continuous variables, we calculated means and standard deviations, with comparisons between control and standardized cohorts performed using *t* tests (PROC GLM in SAS). In some cases, designed contrasts were used to examine differences within larger groups more finely. A time-series analysis was also performed that correlates the trend in taper length for both methadone and lorazepam over the time of the project.

## RESULTS

This study included 49 patients. The control group had 24 patients: 17 patients received both a methadone and lorazepam taper, whereas 7 received only a methadone taper. The standardized protocol group had 25 patients: 16 patients received both methadone and lorazepam taper, 6 patients received only a methadone taper, and 3 patients received only a lorazepam taper. Table 1 presents the relevant demographic variables. No statistical differences were noted between the control and standardized protocol groups other than a younger age in the methadone control group (7.0 ± 10.5 months) compared with the methadone standardized protocol group (26.6 ± 42.2 months; *P* value = 0.0326).

**Table 1.**
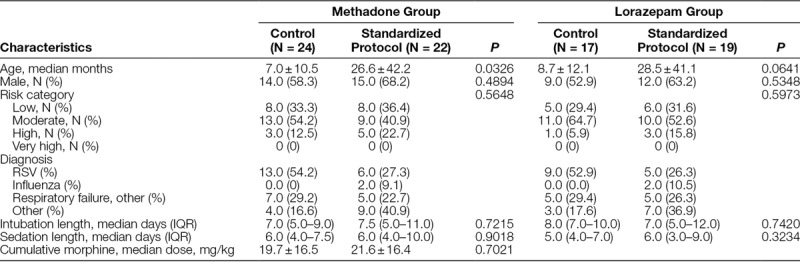
Patient Demographics

### Methadone Results

**Fig. 1.**
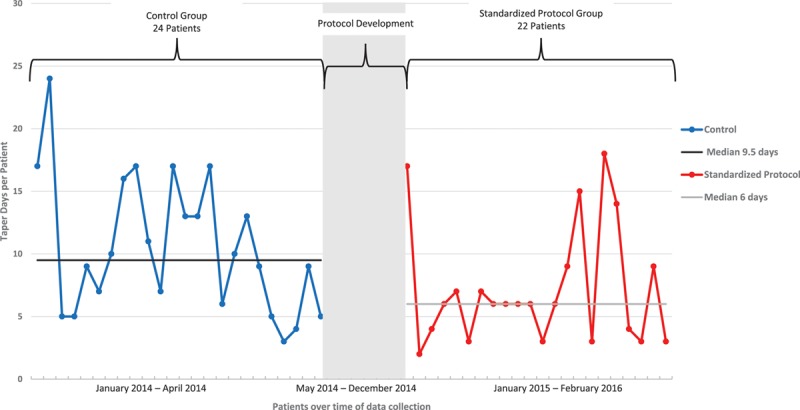
Reduction in median taper length—methadone. Control group is designated in blue by individual patients with median taper days of 9.5. Implementation period is designated in grey when the protocol was developed. Standardized protocol group is designated in red by individual patients with improved median taper days to 6.

Table 2 displays the methadone taper results. The overall median taper length in the control group was 9.5 (IQR, 5.5–14.5) days compared with 6.0 (IQR, 3.0–9.0) days in the standardized protocol group (*P* = 0.0145; Fig. 1 and Table 2). After stratification of patients by risk category, this reduction in taper length remained for both the low and moderate risk categories. Fewer patients in the standardized protocol group were discharged on methadone (31.5%) than in the control group (50%). Although this was not statistically significant, the trend was toward fewer patients being discharged home on methadone (*P* = 0.2109). No patient in either the control or standardized protocol group required naloxone administration for reversal.

**Table 2.**
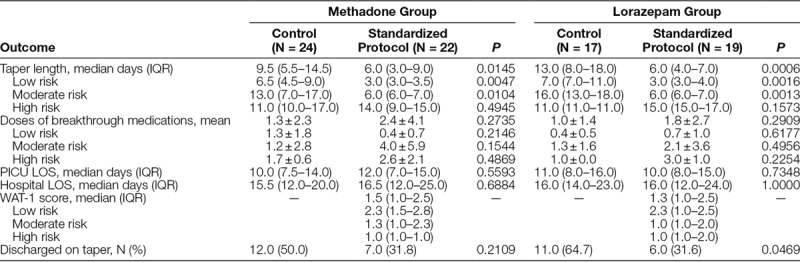
Methadone and Lorazepam Outcomes

### Lorazepam Results

**Fig. 2.**
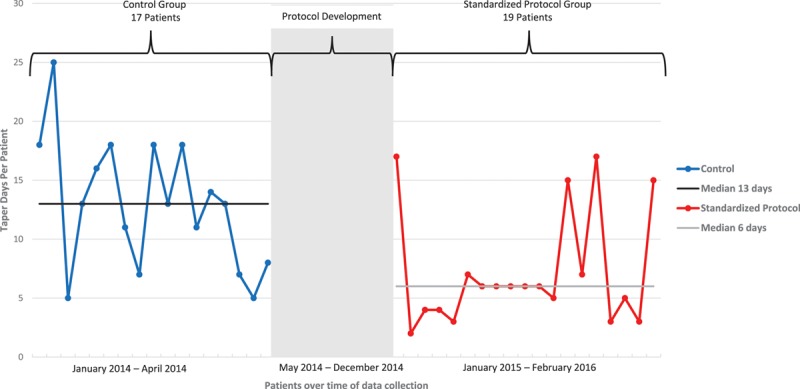
Reduction in median taper length—lorazepam. Control group is designated in blue by individual patients with median taper days of 13. Implementation period is designated in grey when protocol was developed. Standardized protocol group is designated in red by individual patients with improved median taper days to 6.

The lorazepam taper results are also displayed in Table 2. The overall median taper length in the control group was 13.0 (IQR, 8.0–18.0) days compared with 6.0 (IQR, 4.0–7.0) days in the standardized protocol group (*P* = 0.0006; Fig. 2 and Table 2). This reduction in taper length in the standardized protocol group remained for patients stratified to both the low and moderate risk categories. Fewer patients were discharged on lorazepam in the standardized protocol group than the control group (31.6% and 64.7%; *P* = 0.0469). No patient in either the control or standardized protocol group required flumazenil administration for reversal.

### Survey Results

**Fig. 3.**
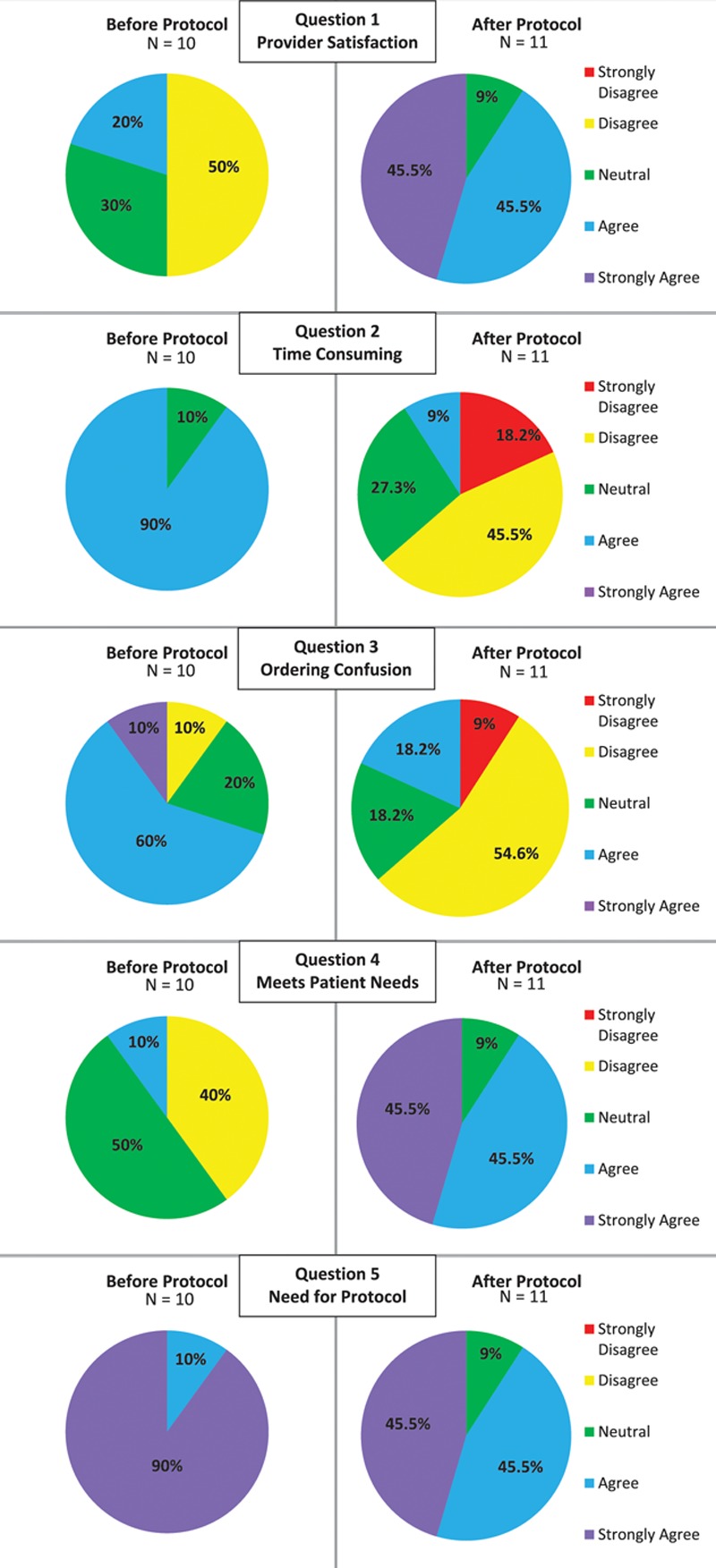
Provider satisfaction survey. Pie graphs used to depict provider satisfaction before and after implementation of the standardized protocol.

All pediatric hospitalists and pediatric intensivists received the preimplementation survey (n = 12) and postimplementation surveys (n = 13). Response rates were 83.3% and 84.6%, respectively. Results are shown in Figure 3. Provider satisfaction with withdrawal management improved after the implementation of the standardized protocol. Additionally, physicians found the standardized protocol to be less time consuming and less confusing to implement than the previous prescribing practices. Physicians who responded also found the standardized protocol to be more likely to meet the needs of patients.

### Deviations from Protocol

Withdrawal management could deviate from the standardized protocol at any time per the prescriber’s clinical judgment. Pharmacists monitored patients and held the taper on the current step, per protocol, for 1 additional day if patients were exhibiting signs of unmanaged withdrawal including the use of 2 or more doses of breakthrough medications or a WAT-1 score greater than or equal to 3 in the previous 24 hours. In the methadone group, 22.7% of patients (5/22) had a deviation from the standard protocol. One patient required an increase in methadone dose, 2 patients were initiated on a lower starting dose per physician discretion, 1 patient’s taper was shortened to facilitate discharge, and 1 patient had the dose of methadone decreased due to concerns for excess sedation. In the lorazepam group, 6 of 19 patients (31.6%) required deviation from the standard protocol. Of these, 2 patients were initiated at a lower dose per prescriber discretion. Two patients appeared to have excess sedation, and the dose was decreased. One patient required an increase in lorazepam dose for withdrawal symptoms, and 1 patient was tapered more quickly to facilitate discharge.

## DISCUSSION

Although previous research has evaluated the results of pharmacist-managed opioid withdrawal, this study is the first report of pharmacist-managed withdrawal tapers for both benzodiazepine and opioid withdrawal in children. We found a statistically significant difference in taper length for both the methadone and lorazepam groups that favored the standardized protocol. Steineck et al.^14^ showed similar results in their study; a pharmacist-managed methadone taper reduced the average taper length from 24.7 days to 15 days. However, research by Johnson et al.^17^ did not find a difference in taper length between pharmacist-managed and physician-managed children with methadone use for iatrogenic opioid withdrawal, although pharmacist-managed patients had fewer elevated withdrawal scores.^17^ Additionally, Abdouni et al.^25^ reported a reduction in methadone taper length from 15.3 days to 9.5 days with the use of a standardized withdrawal guideline, though this was not a pharmacist-managed protocol.^25^

When evaluating the median taper length by risk category, both the low risk and moderate risk groups showed a reduction in taper length with the protocol. The high-risk groups for both methadone and lorazepam demonstrated an increase in taper length with the protocol. However, there were fewer patients in the high-risk groups (methadone control n = 3, methadone protocol n = 5, lorazepam control n = 1, lorazepam protocol n = 3). After closer evaluation, mean taper days were similar (12.67 days versus 13 days) for methadone.

There was a trend toward increased utilization of intravenous morphine and/or lorazepam for breakthrough withdrawal symptoms in the standardized protocol group compared with the control group although the difference was not statistically significant. Although an increase in breakthrough medication use might be correlated with increased withdrawal symptoms, the higher utilization was possibly secondary to several factors including availability of these medications, introduction of the WAT-1 scoring tool, and heightened awareness of withdrawal symptoms by nursing and providers. In the standardized protocol, these breakthrough medications are a required component upon ordering a withdrawal taper to ensure appropriate and timely management of withdrawal symptoms. Also, this study coincided with institutional introduction of the WAT-1 scoring tool, a widely utilized scoring system to assess withdrawal symptoms in pediatric iatrogenic withdrawal.^26^ With the introduction of the WAT-1 scoring tool, nursing staff became more aware of withdrawal signs and symptoms, enabling them to readily recognize withdrawal and therefore quickly manage it appropriately with breakthrough medications. The doses of breakthrough medications were standardized in the protocol group, whereas the doses were at the discretion of the prescriber in the control group. The doses used in the control group were not evaluated, but this may have further impacted results.

Many different approaches to dosing methadone have been reported. This study chose a risk-based titration protocol with fixed-dose methadone, as it was most similar to previous prescribing practices at the study institution. A recent study evaluated patients who were rapidly converted from intravenous opioids to methadone compared with those who were slowly converted.^27^ The patients who had a successful rapid conversion to methadone received a methadone:fentanyl conversion ratio of approximately 2.5:1. Shaheen et al.^28^ demonstrated that conversion calculations are widely variable and require clinical judgment and patient-specific modifications to be used safely. A review by Johnson et al.^11^ evaluated weight-based and formula-based methadone conversions and concluded that patients should be initiated on the lowest methadone dose possible and then titrated based on the patient’s response to minimize adverse effects. In the standardized protocol presented here, we used fixed-dose methadone, although modification was allowed at any time based on patient-specific factors.

Several patient safety concerns were identified and rectified during the study. Review of the control group revealed potentially excessive length of tapers. Implementation of this standardized protocol has demonstrated a reduction in prolonged and potentially unnecessary withdrawal tapers. We identified discharge prescribing errors both pre- and postprotocol implementation. Based on the observed data, pharmacists are now involved in all hospital discharges for children on opioid and benzodiazepine withdrawal tapers. Pharmacists review the accuracy of orders upon discharge. They provide the patient and their family with detailed instructions and a calendar to help ensure the taper is completed appropriately at home. With a decrease in taper length, we hope to see a further reduction in discharges with these medications to help avoid these potential errors in the future.

There were limitations to this study. The standardized protocol cohort had older children compared with the control, which may have affected results as withdrawal symptoms and response to treatment can vary by age. We attribute this difference in age to variability in viral illnesses during the season of data collection. The control cohort contained a large number of infants with bronchiolitis, whereas the standardized protocol patients had a variety of diagnoses. The variability in disease states also contributed to the discrepancy in date ranges for the standardized protocol and control groups. The intent was to enroll a similar number of patients in both groups over the same duration of time. However, the standardized protocol cohort required 14 months to enroll a similar number of patients as compared with the control group, which had occurred over a 4-month period. This longer enrollment period was also likely due to differences in the severity of seasonal viruses as previously discussed. We utilized a standard nurse-driven sedation protocol during both the retrospective control and prospective standardized protocol periods; therefore, differences in sedation practices over time were not felt to be a contribution to the difference in patient enrollment between groups. We did not evaluate the potential effects of other medications, such as dexmedetomidine or ketamine, on withdrawal symptoms. Deviation from the standard protocol occurred in 22.7% of the methadone patients and 31.6% of the lorazepam patients. These rates of protocol deviation were similar to that published by Abdouni et al.^25^ who reported 67.1% compliance rate with their standard withdrawal guideline. Other limitations of the study include small sample size, lack of severity of illness scoring, no cost analysis, and nonwithdrawal discharge barriers affecting the length of stay.

## CONCLUSIONS

The implementation of a standardized pharmacist-driven methadone and lorazepam taper protocol resulted in a decrease in taper length. Also, providers reported satisfaction with the standardized protocol. Further evaluation of this taper involving a larger patient population is needed.

## ACKNOWLEDGMENTS

The authors gratefully acknowledge the pediatric intensive care faculty and pediatric pharmacy staff for their support and participation in this project. The authors thank Linda Oyen, RPh, pediatric pharmacy supervisor, for her leadership and direction of this protocol and Daniele Heyn, RN, CNP, for her work on implementing the WAT-1 scoring tool. The authors also thank Saquib Lakhani, MD, FAAP, and Benson Hsu, MD, MBA, FAAP, for their article guidance.

## DISCLOSURE

The authors have no financial interest to declare in relation to the content of this article.

## Supplementary Material
