# Supplementary material for: Shortened Taper Duration after Implementation of a Standardized Protocol for Iatrogenic Benzodiazepine and Opioid Withdrawal in Pediatric Patients: Results of a Cohort Study
Source: Pediatr Qual Saf. 2018 May 18;3(3):e079. doi: 10.1097/pq9.0000000000000079 (PMC6132810; doi:10.1097/pq9.0000000000000079)
Supplement: Supplementary file 1 [file pqs-3-e079-s001.pdf]

Sanford Children's Iatrogenic Withdrawal Management Protocol

Low Risk Patients (<5 days opioid/BNZ infusion)[a]		
**Day**	**Methadone Dosing**	**Lorazepam Dosing**
1	0.05 mg/kg/dose PO Q 8 hours for 3 doses	0.05 mg/kg/dose PO Q 6 hours for 4 doses
2	0.05 mg/kg/dose PO Q 12 hours for 2 doses	0.05 mg/kg/dose PO Q 8 hours for 3 doses
3	0.05 mg/kg/dose PO Q 24 hours for 1 dose	0.05 mg/kg/dose PO Q 12 hours for 2 doses

Moderate Risk Patients (5-9 days opioid/BNZ infusion)		
**Day**	**Methadone Dosing**	**Lorazepam Dosing**
1	0.1 mg/kg/dose PO Q 8 hours for 3 doses	0.1 mg/kg/dose PO Q 6 hours for 4 doses
2	0.08 mg/kg/dose PO Q 8 hours for 3 doses	0.1 mg/kg/dose PO Q 8 hours for 3 doses
3	0.06 mg/kg/dose PO Q 8 hours for 3 doses	0.08 mg/kg/dose PO Q 8 hours for 3 doses
4	0.04 mg/kg/dose PO Q 8 hours for 3 doses	0.06 mg/kg/dose PO Q 8 hours for 3 doses
5	0.04 mg/kg/dose PO Q 12 hours for 2 doses	0.04 mg/kg/dose PO Q 8 hours for 3 doses
6	0.04 mg/kg/dose PO Q 24 hours for 1 dose	0.04 mg/kg/dose PO Q 12 hours for 2 doses

High Risk Patients (≥10 days opioid/BNZ infusion or cumulative morphine dose of 60-100 mg/kg or equivalent)		
**Day**	**Methadone Dosing**	**Lorazepam Dosing**
1	0.1 mg/kg/dose PO Q 6 hours for 8 doses	0.1 mg/kg/dose PO Q 6 hours for 12 doses
2		
3	0.1 mg/kg/dose PO Q 8 hours for 6 doses	
4		0.1 mg/kg/dose PO Q 8 hours for 6 doses
5	0.08 mg/kg/dose PO Q 8 hours for 6 doses	
6		0.08 mg/kg/dose PO Q 8 hours for 6 doses
7	0.06 mg/kg/dose PO Q 8 hours for 6 doses	
8		0.06 mg/kg/dose PO Q 8 hours for 6 doses
9	0.04 mg/kg/dose PO Q 8 hours for 6 doses	
10		0.04 mg/kg/dose PO Q 8 hours for 6 doses
11	0.04 mg/kg/dose PO Q 12 hours for 4 doses	
12		0.04 mg/kg/dose PO Q 12 hours for 4 doses
13	0.04 mg/kg/dose PO Q 24 hours for 2 doses	

Very High Risk Patients (≥28 days opioid/BNZ infusion or cumulative morphine dose of >100 mg/kg or equivalent)		
**Day**	**Methadone Dosing**	**Lorazepam Dosing**
1	0.2 mg/kg/dose PO Q 6 hours for 8 doses	0.1 mg/kg/dose[b] PO Q 4 hours for 6 doses
2		0.1 mg/kg/dose PO Q 6 hours for 8 doses
3	0.2 mg/kg/dose PO Q 8 hours for 6 doses	
4		0.1 mg/kg/dose PO Q 8 hours for 6 doses
5	0.17 mg/kg/dose PO Q 8 hours for 6 doses	
6		0.09 mg/kg/dose PO Q 8 hours for 6 doses
7	0.14 mg/kg/dose PO Q 8 hours for 6 doses	
8		0.08 mg/kg/dose PO Q 8 hours for 6 doses
9	0.11 mg/kg/dose PO Q 8 hours for 6 doses	
10		0.07 mg/kg/dose PO Q 8 hours for 6 doses
11	0.08 mg/kg/dose PO Q 8 hours for 6 doses	
12		0.06 mg/kg/dose PO Q 8 hours for 6 doses
13	0.05 mg/kg/dose PO Q 8 hours for 6 doses	
14		0.05 mg/kg/dose PO Q 8 hours for 6 doses
15	0.05 mg/kg/dose PO Q 12 hours for 4 doses	
16		0.05 mg/kg/dose PO Q 12 hours for 4 doses
17	0.05 mg/kg/dose PO Q 24 hours for 2 doses	

[a] Patients receiving <5 days of continuous infusion are not expected to experience withdrawal. Do not treat unless signs/symptoms occur.
[b] May increase to 0.2 mg/kg/dose if desired; then decrease by 15% each time instead of 10%

If 2 or more doses of withdrawal morphine IV as-needed, OR 2 or more doses of withdrawal lorazepam IV as-needed are required in a 24 hour period, OR a WAT-1 score of greater than or equal to 3: consider remaining on current step (recommended) or back up one step.

- Morphine 0.05 mg/kg IV every 2 hours as-needed for withdrawal symptoms
- Lorazepam 0.05 mg/kg IV every 2 hours as-needed for withdrawal symptoms
- Breakthrough doses will be switched to every 4 hours when the patient is transferred to the floor

This protocol was developed for use at Sanford Health. Before using this protocol or associated documents, it is recommended to conduct independent clinical and operational review. Further, each situation is unique and this protocol may need to be altered on a case-by-case basis. No specific results are guaranteed from the use of this protocol. Last reviewed January, 2018.
